# The Effectiveness of Different Treatment Modalities of Cutaneous Angiosarcoma: Results From Meta-Analysis and Observational Data From SEER Database

**DOI:** 10.3389/fonc.2021.627113

**Published:** 2021-02-25

**Authors:** Siwei Bi, Shanshan Chen, Beiyi Wu, Ying Cen, Junjie Chen

**Affiliations:** ^1^ Department of Burn and Plastic Surgery, West China Hospital, Sichuan University, Chengdu, China; ^2^ West China School of Medicine, Sichuan University, Chengdu, China

**Keywords:** cutaneous angiosarcoma, SEER database, treatment modalities, meta-analysis, clinical efficacy, 5-year death rate, overall survival, cancer-specific survival

## Abstract

**Introduction:**

Cutaneous angiosarcoma (cAS) is an aggressive vascular tumor that originates from vascular or lymphatic epithelial cells. To date, the cAS literature has been limited in a small number with single-center experiences or reports due to its rarity and the optimal treatment strategy is still in dispute. This study aimed to conduct a systematic review and compare the effect of available treatments retrieved from observational studies and Surveillance, Epidemiology, and End Results (SEER) program.

**Methods:**

The authors performed a systematic review in the PubMed, Embase and MEDLINE database identifying the researches assessing the treatment for cAS patients. Clinical and treatment information of patients who had been diagnosed with a primary cAS were also obtained from the SEER program.

**Results:**

Thirty-two studies were eligible but only 5 of which with 276 patients were included in meta-analysis since the unclear or unavailable information. The risk ratio of 5-year death for surgery, surgery with radiotherapy and surgery with chemotherapy were 0.84, 0.96, and 0.69. Meanwhile, in SEER database, there are 291 metastatic and 437 localized patients with cAS. The localized patients receiving surgery showed a significantly worse overall survival result when compared with the surgery combined with RT: hazard ratio: 1.6, 95% confidential interval: 1.05, 2.42, P = 0.03.

**Conclusion:**

In conclusion, our study provided a detailed picture of the effectiveness of present treatments for localized and metastatic cAS patients. The CT could be inappropriate in localized patients. For metastatic patients, the surgery combined RT was recommended compared with surgery alone since its enhanced OS prognosis. Yet, more novel-designed clinical trials with specific targeted populations and rigorous conducting are needed for a solid conclusion on which would be a better treatment strategy.

## Introduction

Angiosarcomas are a group of vascular malignant tumors that are relatively rare and account for 1-2% of all soft tissue sarcomas (1). With an extremely poor prognosis, patients with angiosarcomas always ending within a year (2). They originate from vascular or lymphatic epithelial cells and can arise in various locations of the body (3, 4). About 60% of angiosarcomas present as cutaneous angiosarcomas (cAS) involving the head and neck predominantly. Others can exist in visceral organs, bones, and other soft tissues (4, 5). Multiple factors are proved to affect the survival rates of cAS, including age, tumor size, tumor site and so on (6).

The prognosis of cAS is relatively poor with a 5-year survival rate ranging from 26% to 51% (6, 7). There are many treatment options for cAS (8, 9), including surgery (10, 11), radiotherapy (RT) (12), chemotherapy (CT) (13), targeted therapy (14, 15) and more recently, immunotherapy (IT) (16). Mainstay therapy remains surgery with adjuvant RT (9). However, with the presence of new effective strategies, the treatment choice for cAS patients could be controversial. Besides, limited literature focused on the possible prognostic significance of treatments on different groups of patients such as metastatic or localized, which would be confusing in clinical practice.

Surveillance, Epidemiology, and End Results (SEER) Program of the National Cancer Institute (17) was initiated in 1973. SEER has now gained enough data that clinical and descriptive characteristics of uncommon tumors can be described at a population level. Based on the clinical characteristics, survival outcomes and corresponding therapy information retrieved from SEER program, we compared the therapeutic effect of different treatments of cAS patients. Moreover, we performed a systematic review and meta-analysis to summarize the previous observational studies evaluating the efficacy of different therapies in treating cAS, through which, independent results of the previous studies could be synthesized.

## Methods

### Meta-Analysis: Data Sources and Search Strategy

The following English databases were searched systematically: PubMed, EMBASE and Medline Database with: (cutaneous angiosarcoma [Title/Abstract]) AND (treatment [Title/Abstract]). Only English articles published up to the searching date: 2020.5.17 were included. Reference lists of primary articles were reviewed for more literature.

### Meta-Analysis: Inclusion Criteria and Study Selection

Inclusion criteria are as follows: 1) sufficient data including age, tumor size, tumor site, treatments were provided in a full-length article; 2) study design: prospective or retrospective cohort trials; 3) Outcome measurements: survival rate and corresponding follow up duration. Meanwhile, we excluded studies without enough data for effect sizes calculation or any case reports, review articles, letters, or communications. Two reviewers (SWB, SSC) independently went through the titles and abstracts. A senior reviewer (JJC) would be consulted if any differences exist.

### Meta-Analysis: Data Extraction and Quality Assessment

By the Cochrane Collaboration for Systematic Reviews guidelines (18), this process was performed separately by two reviewers (SWB, SSC). Relevant data from the eligible studies were extracted including the 1st author’s name, the published year, the number of participants, gender proportion, median age, tumor site, tumor size, tumor grade, tumor presentation, average follow-up time, treatment, and outcome measurements. The methodologic quality of each study was evaluated according to the assessment of the Newcastle–Ottawa scale which comprises three categories, including the selection of the study population. comparability of the groups, and ascertainment of the exposure or outcomes. Each parameter consists of a subcategorized questionnaire based on selection, comparability, and outcomes (19, 20). Two of the authors (SWB, SSC) independently scored the questionnaire for each included study following the user manual of the Newcastle–Ottawa scale.

### SEER Database: Selection of Population Data and Outcomes

We chose the SEER 18 database which includes cases recorded between 1973 and 2015 spanning 18 different US geographic areas. The clinical data of patients who were diagnosed with cAS were obtained from the SEER Program. cAS was defined by combining the International Classification of Diseases for Oncology, 3rd edition (ICD-O-3) morphological code 9120/3 and 9170/3, which stands for hemangiosarcoma and lymphangiosarcoma, and topographical codes: C44.0-9. The other variables were included such as age at diagnosis, sex, tumor grade, tumor site, tumor size, SEER historic stage, treatment modalities and survival outcomes. For the SEER historic stages, “local,” “regional,” and “distant” were used as the End Results Group of National Cancer Institute (NCI).

### Statistical Analysis

A single group meta-analysis was performed and results were presented with 95% confidence interval (CI). Studies were then pooled together as appropriate with two-sided P < 0.05 considered as statistically significant. The authors calculate the Q-statistic (21) for testing heterogeneity among studies, and P < 0.05 was considered as significant too. The authors selected the results with the fixed-effects model if the included studies were homogenous with P > 0.05; otherwise, the random-effects model results would be picked on. The I^2^ statistic (21) was also calculated to efficiently test for the heterogeneity, with I^2^ < 25%, 25%–75%, and > 75% to represent a low, moderate and high degree of heterogeneity, respectively. We conducted a subgroup analysis to detect the source of heterogeneity furtherly based on the different treatment strategies.

On the other hand, for the SEER database analysis, Kaplan-Meier curves were used to illustrate the overall survival (OS) and cancer-specific survival (CSS) probabilities for the selected patients grouped by different therapies. The univariate and multivariate cox proportional hazards regression models were performed using the log-rank test. Predictors for the multivariate model were the factors identified as statistically significant (*P* value <0.05) in univariate analysis. Moreover, the authors plotted the trends in the management of patients with cAS with linear regression analysis. All the analysis and plots were generated using R 3.6.2 with packages (22–26): “gemtc,” “rjags,” “dmetar,” “survival,” “survminer,” and “ggplot2”.

## Results

### Meta-Analysis: Eligible Studies Identification

**Figure 1:**
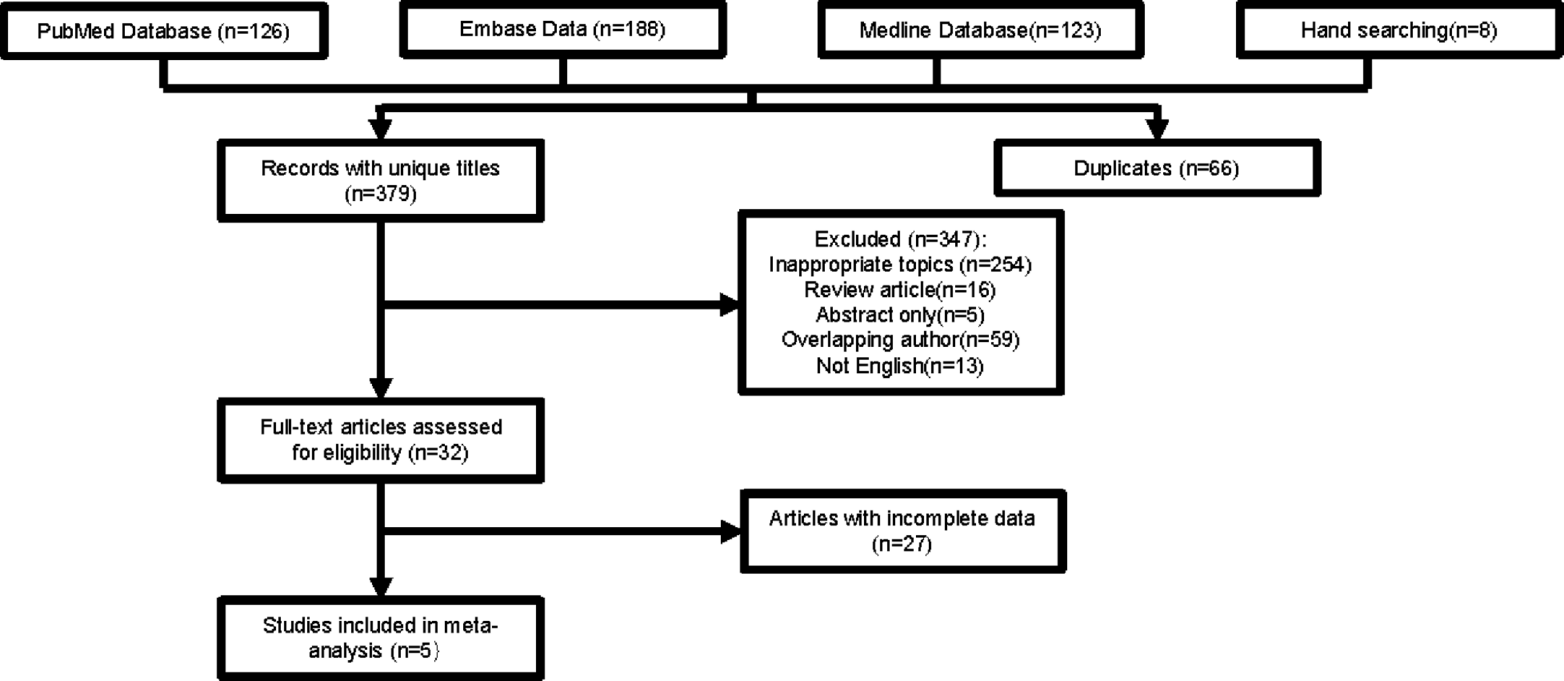
Study selection process.

As shown in **Figure 1**, 445 studies were chosen from databases for further screening. We excluded 66 duplicated articles and 347 other articles because of inappropriate topics (n=254), review articles (n=16), lack of full text (n=5), overlapping author (n=59), and not English (n=13). After assessing articles with full text, 32 studies were selected in total. A large number of studies were short of precise data for a specific treatment arm. In the end, five studies with 276 participants were included for the meta-analysis.

### Meta-Analysis: Characteristics of Selected Studies

The clinical characteristics of both selected observational studies and SEER population were summarized in **Table 1**. The detailed characteristics of 32 included studies are shown in **Supplementary Files**. The sample size ranged from 5 to 421 with a median of 44 and 1414 participants in total. Participants of 17 studies were divided into two groups by tumor size. Twenty-eight studies involved information about tumor site and 13 studies involved tumor grade. The majority of studies focused on the efficacy of surgery and RT (n=22).

**Table 1:** Patient demographics and tumor characteristics for cutaneous angiosarcomas summarized from published literature and SEER database.

	Published literatures	SEER
Sex		
Male	916 (64.8%)	435 (48.4%)
Female	498 (35.2%)	464 (51.6%)
Age		
10–39	72.1 ± 5.15^a^	14 (1.6%)
40–49		31 (3.4%)
50–59		70 (7.8%)
60–69		177 (19.7%)
70–79		280 (31.1%)
80+		327 (36.4%)
Race		
White	–	791 (88.0%)
Black	–	42 (4.6%)
Other	–	49 (5.5%)
Unknown	–	17 (1.9%)
Average follow up (months)^b^	112.9	43.7
Size		
Tumor size ≤5	525 (37.1%)	11 (1.2%)
Tumor size >5	432 (30.6%)	357 (39.7%)
NA/Not reported	457 (32.3%)	531 (59.1%)
Sites		
Scalp/neck/head	721 (51.0%)	345 (39.2%)
Face	367 (26.0%)	211 (21.7%)
Trunk/limb	41 (2.9%)	326 (37.1%)
Unspecific site Unknown	152 (10.7%) 133 (9.4%)	17 (1.9%) -
Histologic grade		
Grade I	–	54 (6.0%)
Grade II	–	83 (9.2%)
Grade III	–	138 (15.4%)
Grade IV	–	128 (14.2%)
Unknown	–	496 (55.2%)
SEER historic stage		
Localized	–	437 (51.6%)
Distant	–	291 (34.3%)^c^
Unstaged	–	119 (14.1%)

a : Mean ± Standard deviation.

b : Mean value of longest follow-up time from each study.

c : There are 62 distant and 229 regional patients.

SEER, Surveillance, Epidemiology, and End Results program; NA, not available.

### Meta-Analysis: Summary of Prognosis Results in Eligible Studies

The summary of prognosis parameters: 2-, 3-, 5-, 10-year survival rate, disease-free interval (DFI), mean survival time and 3, 5-year regression free survival (RFS) are shown in **Table 2** severally. The 5-year survival rate in patients receiving surgery was 12.5%–46.9%. In patients treated with RT, the 5-year survival rate was 0%–16.7%. Surgery treatment had the highest 3-year survival rate which was close to that of surgery combined with RT (60.2% and 58.4% respectively). Besides, with the follow-up time extending, the survival rate decreased, especially from 3-year to 5-year: for surgery, from 60.2% to 12.5%–46.9%; for RT, from 33.3% to 0%–16.7%; for surgery and RT, from 58.4% to 0%–33.3%.

**Table 2:** Summary results of prognosis in included studies.

Therapy type	N	2 year-survival rate (%)	3 year-survival rate (%)	5 year-survival rate (%)	10 year-survival rate (%)	3 year-RFS (%)	5 year-RFS (%)	DFI (month)	Mean survival time (month)	First author
Surgery	7–48		60.2	12.5–46.9	14.3	59.8	25–39.9		12.9	Perez, MC (27); Matsumoto, K (28); Holden, CA (29); Zhang, Y (30)
RT	7–45	29	33.3	0–16.7					10.8	Perez, MC (27); Matsumoto, K (28); Holden, CA (29)
CT	2			0					2	Matsumoto, K (28)
Surgery and RT	3–57	66.7	58.4	0–33.3		27.9	0–27.9	42.8	31.3	Perez, MC (27); Matsumoto, K (28); Holden, CA (29); Zhang, Y (30); Barttelbort, SW (31)
Surgery and CT	8–22			0–14			7		11.9	Matsumoto, K (28); Zhang, Y (30)
Surgery, RT and CT	7–22			13.3–15			0		17.1	Matsumoto, K (28); Zhang, Y (30)
RT and CT	13			61.5					9.3	Matsumoto, K (28)

N, Number of patients enrolled in each study; CT, chemotherapy; RT, radiotherapy; RFS, recurrence-free survival; DFI, disease-free interval.

### Meta-Analysis: Results for Death Rate

**Figure 2:**
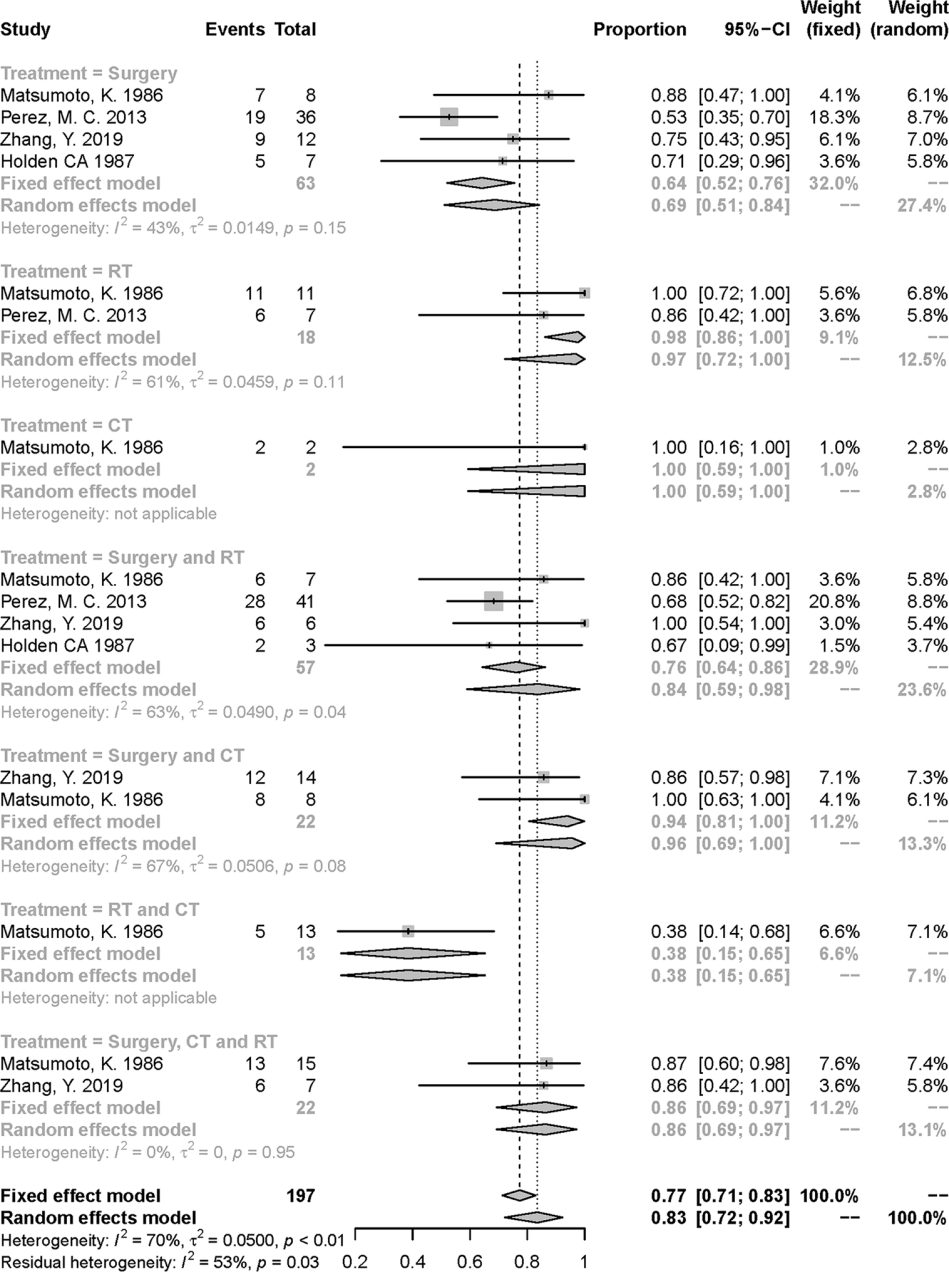
Meta-analysis of 5-year over-all death rate in included studies. RT, radiotherapy; CT, chemotherapy.

Similarly, in **Figure 2**, the treatment of RT and CT had the lowest 5-year death rate followed by the treatment of surgery [risk ratio (RR):0.38, 95% confidential interval (CI) = 0.15–0.65; 0.69, 95% CI = 0.51–0.84; respectively]. However, the small number of patients in RT and CT group should be noted. The heterogeneity was in a moderate degree in the pooled effect (I^2^ = 70%, *P* < 0.01) and subgroups of several treatments (**Figure 2**). We also tried to conduct a subgroup analysis to detect the source of heterogeneity furtherly based on other various factors including metastasis condition, age, tumor size, and tumor site, but failed since enrolled articles were lack of appropriate data.

### Meta-Analysis: Study Quality of Included Studies

The summary quality assessment of the 32 included studies was illustrated in **Supplementary Files**. We assigned scores of 0–3, 4–6, and 7–9 on the Newcastle-Ottawa scale for the low, moderate and high quality of studies, respectively. The 32 included studies showed the mean quality score was 7 out of 9. In the 5 enrolled studies, three studies reached 8 and two studies were ranked as 7.

### SEER Database: Characteristics of the Population

In **Table 1**, we retrieved 899 cAS patients from the SEER database where 435 patients were male and 464 were female. Interestingly, the ratio of patients with tumor size more than 5 cm versus less than 5cm was exponentially larger than that in published literature data. As for the tumor site, a larger proportion of tumors were documented in the trunk/limb when comparing the SEER data with the published literature data. There are 62 distant and 229 regional patients grouping as distant patients in the following analysis. The number of patients receiving surgery, surgery and RT, surgery and CT, surgery and RT and CT, were 389 (43%), 173 (19%), 61 (7%), and 54 (6%) respectively. There are 108 patients with no treatments recorded (12%).

### SEER Database: Factors Influencing the OS and CSS

In the univariate analysis, sites of face (*P* value < 0.01) and trunk/limb (*P* value < 0.01) were predictors of both OS and CSS. Ages (*P* value < 0.01), size (*P* = 0.03), black race (*P* value < 0.01), localized stage (*P* value < 0.01), tumor grades (*P* value < 0.05) except grade II (*P* value= 0.54) were all significant predictors of OS. Age (*P* value < 0.05), sex (*P* value < 0.01), and SEER historic stage (*P* value < 0.05) were predictors for CSS (**Supplementary Table 3**). The multivariate models conducted for both OS and CSS included all significant predictors in univariate analysis (**Supplementary Table 4**). We also included the treatment modalities as covariates. All age groups were independently correlated with OS in localized patients. Sites of face and trunk/limb were found to reduce the OS and CSS in localized patients and the OS in metastatic patients when compared with the reference groups (*P* value < 0.05).

### SEER Database: Effectiveness and Trends of Different Treatment Modalities

**Figure 3:**
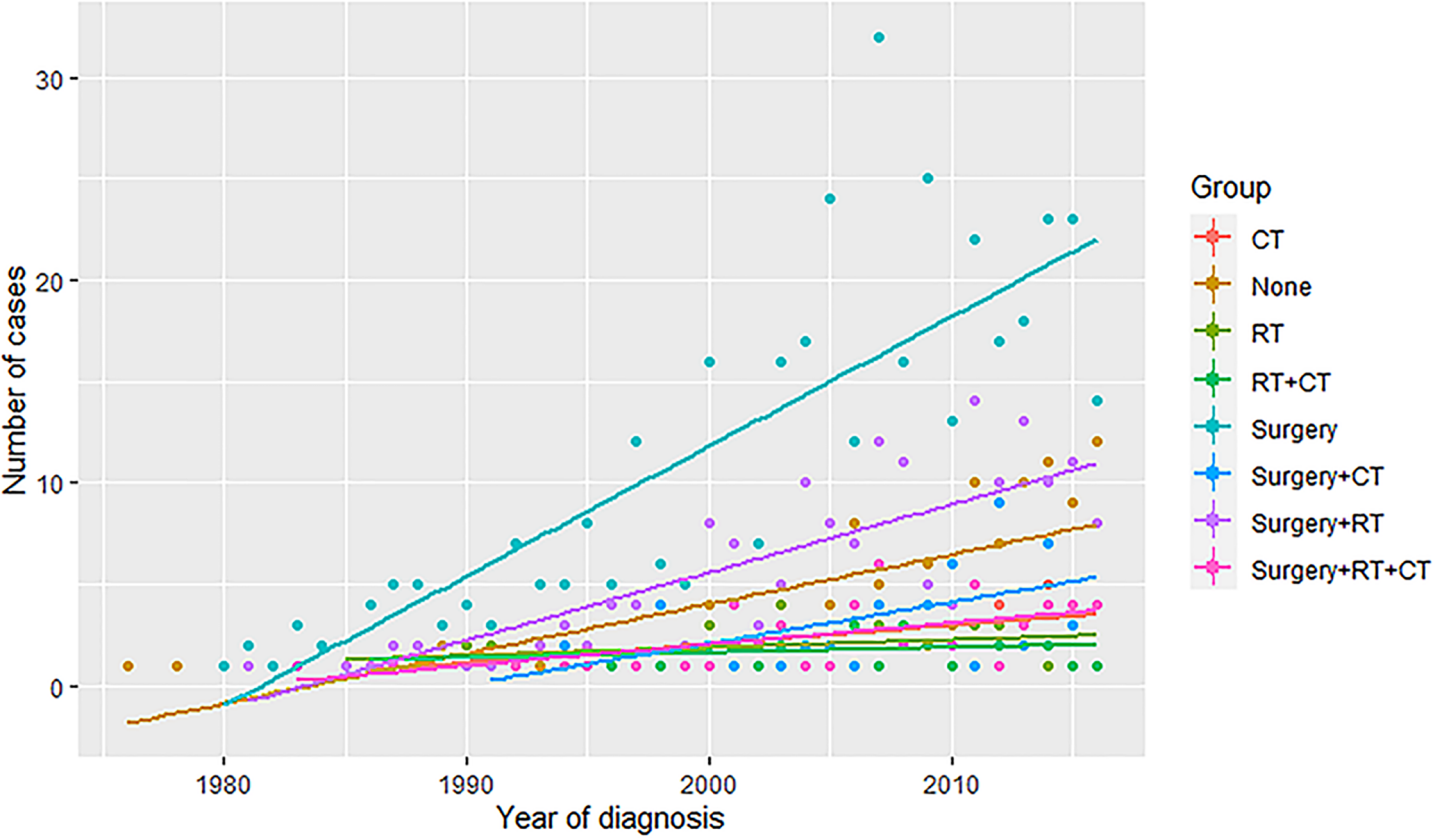
The trends of therapies based on the number of patients who received the same therapy each year. RT, radiotherapy; CT, chemotherapy.

For a more accurate illustration of the efficacy of different treatment modalities, the multivariate cox regression analysis was performed in which the hazard ratio of OS and CSS were adjusted by the significant factors in the univariate analysis. (Full results were shown in **Supplementary Table 4**). As shown in **Table 3**, the patients were stratified into localized and metastatic groups. Compared with the surgery with RT group, both localized and metastatic patients treated with CT showed significantly worse outcomes in OS and CSS, while the surgery and CT group and surgery and CT and RT group showed significantly worse OS only in localized patients. Particularly, the surgery alone was associated with a higher hazard for OS in metastatic patients compared with the surgery with RT group [hazard ratio (HR): 1.6; 95% CI: (1.05, 2.42); *P* value: = 0.03]. In **Figure 3**, we plotted the trends of therapies based on the number of patients who received the same therapy each year. Surgery is the most commonly used therapy followed by surgery together with radiotherapy.

**Table 3:** Multivariate cox proportions hazards models for overall survival (OS) and cancer-specific survival (CSS) in SEER patients with cAS.

Treatment modality	Localized						Metastatic					
	OS			CSS			OS			CSS		
	HR	95% CI	P value	HR	95% CI	P value	HR	95% CI	P value	HR	95% CI	P value
Surgery and RT	Ref											
CT	**3.6**	**(1.95,6.62)**	**<0.01**	**3.16**	**(1.17,8.53)**	**0.02**	**4.17**	**(2.05,8.46)**	**<0.01**	**3.53**	**(1.24,10.02)**	**0.02**
None	1.72	(1.01,2.91)	0.05	1.83	(0.83,4.05)	0.13	**4.36**	**(2.3,8.25)**	**<0.01**	**6.78**	**(2.77,16.59)**	**<0.01**
RT	1.62	(0.94,2.77)	0.08	0.9	(0.36,2.24)	0.81	1.61	(0.83,3.11)	0.16	1.37	(0.52,3.62)	0.53
RT+CT	1.61	(0.82,3.15)	0.16	1.17	(0.44,3.13)	0.75	1.11	(0.54,2.25)	0.78	1.82	(0.76,4.33)	0.18
Surgery	0.99	(0.71,1.4)	0.98	0.62	(0.36,1.07)	0.08	**1.6**	**(1.05,2.42)**	**0.03**	1.15	(0.62,2.15)	0.66
Surgery and CT	**2.25**	**(1.19,4.24)**	**0.01**	1.71	(0.57,5.12)	0.34	1.18	(0.67,2.06)	0.56	1.04	(0.46,2.39)	0.92
Surgery, RT and CT	**1.79**	**(1.03,3.13)**	**0.04**	1.89	(0.88,4.04)	0.1	1.3	(0.72,2.33)	0.38	1.87	(0.91,3.86)	0.09

cAS, cutaneous angiosarcoma; OS, overall survival; CSS, cancer-specific survival; CT, chemotherapy; RT, radiotherapy; HR, hazard ratio; CI, confidential interval.

The significant results (P<0.05) were showed in bold.

## Discussion

Given the limited clinical evidence since the rather low incidence of cAS, the discussion for selecting the optimal treatment modality of cAS was in slow progress. Shin et al. (32) conducted a meta-analysis indicating the factors predisposing poor outcomes for angiosarcoma of the scalp and face. In this study, the only treatment-related result was that surgery, compared with no-surgery patients, the 5-year OS rate of angiosarcomas would significantly increase. They also stated the difficulty of comparing different treatment methods since the absence of data. Other studies focusing on the cAS and angiosarcoma patients in SEER database were all short of treatment modalities information (6, 7). To our knowledge, the present study is the first meta-analysis and SEER database research focused on illustrating the prognosis of the cAS patients based on their treatment modalities and extent of the tumor.

### Localized cAS Patients

For localized patients, the results from the SEER database suggest that the CT could be inappropriate while the necessity of additional RT to surgery remains uncertain. Because CT alone, surgery and CT, surgery and CT and RT showed worse OS results when compared with surgery and RT in the localized patients. The reason could be the intolerance of patients giving a significant proportion of the elderly. What’s more, there were no significant results when comparing surgery alone with surgery and RT in the localized patients for both OS and CSS. Several studies (32, 33) have proven that surgery could enhance prognosis in cAS patients with no stratification of patients. Yet, surgery and RT was widely reported for reducing the risk of local recurrence and improving survival rate in localized patients (34, 35). Guadagnolo et al. (36) demonstrated that non-metastatic patients who underwent surgery and RT have statistically greater local control, OS and disease-specific survival compared with those who received surgery or RT alone. Another review (9) stated that surgery followed by RT is the mainstay of the treatment for localized angiosarcoma. Many reasons would cause this ambiguity. Primarily, the assessment of treatment efficacy should be based on the extent of cAS. Localized cAS patients are prone to receive extensive surgery and with a better prognosis since they are in the early stage of cancer while metastatic patients need more systematic treatment and ended up with a poorer outcome. Thus, any comparison of the treatment regardless of the patients’ condition should be treated with caution. Secondly, most studies, including ours, are limited by the retrospective nature. The doses, frequency and time of RT (before or after the surgery) can vary a lot. There was another trial demonstrating the efficacy of chemoradiotherapy followed by maintenance CT in localized patients with large tumors that are hard to control with surgery and RT (37). Further clinical trials or guidelines may focus more on systematically conducting and delicately grouping of patients.

### Metastatic cAS Patients

Paclitaxel (taxanes) was recommended as the first-line treatment for metastatic cAS patients in (9), which conflicts with our results: metastatic patients treated with CT alone have worse OS and CSS outcomes than the surgery combined with RT group. This discrepancy could derive from the use of different CT drugs since the quickly evolving process of finding new drugs. Doxorubicin-based drugs have been the preferred choice for advanced soft tissue sarcomas earlier (38, 39), which was replaced by paclitaxel nowadays (9, 38–40). Paclitaxel was rigorously assessed in a phase II trial where 30 metastatic angiosarcoma patients enrolled for a median follow-up of 8 months (40), and the result showed the median time to progression was 4 months and the median overall survival was 8 months. One retrospective study from the same institution including 149 metastatic angiosarcoma patients found there were no statistically significant differences in terms of overall survival between weekly paclitaxel and doxorubicin-based therapy (38).

On the other hand, for metastatic patients, we observed a significantly worse OS outcome receiving surgery alone versus surgery and RT only, which provides evidence for surgery and RT use in metastasis patients except for localized patients. As forementioned, the discussion of the treatment modality for metastatic patients should also consider factors including the patients’ tolerance and quality of life and the follow-up duration. Considering the multiple choices of CT drugs, it seems more difficult to reach an agreement. A more systematic treatment modality might be a more reliable choice for metastatic patients based on our findings and current status.

### Booming Treatment Options

According to previous results (9, 41), various drugs could be the second-line treatments for advanced cAS including pazopanib (a tyrosine kinase inhibitor), eribulin mesylate (a microtubule-targeting drug), trabectedin (a histone deacetylase inhibitor), bevacizumab (a vascular endothelial growth factor receptor inhibitor), and propranolol (a beta-blocker). Pazopanib, eribulin mesylate, and trabectedin were firstly published to be effective in treating patients with soft tissue sarcomas (42–44). In later times, a Japanese study showed the potential of pazopanib for the treatment of cAS (45). One prospective clinical study evaluating eribulin mesylate in patients with cAS after taxanes showing a promising response rate (46). Another retrospective study found the 3-month PFS rate was 25% with trabectedin in patients with angiosarcoma (47). Bevacizumab was reported to be effective in treating cAS with a PFS of 6.5 months in a phase II study (48). Notably, propranolol was firstly reported to inhibit the progression of infantile hemangioma (49). Following, several case reports described that the propranolol monotherapy or the combination of propranolol with other chemotherapeutic agents had promising responses in advanced angiosarcoma (50–52).

With the field of cancer immunology growing rapidly, there are also studies linking immune therapy, anti-programmed death ligand-1 (anti-PD-L1), to angiosarcoma treatment. A case report showed a remarkable response in a patient with angiosarcoma with the treatment of anti-PD-L1 (16). Nonetheless, for all the second-line treatments and the immunotherapy, there was not enough evidence to make recommendations for patients with advanced cAS and more prospective studies were needed.

### Limitations

Our review has some limitations. Firstly, due to the rarity of cAS and the unclear classification of the treatment modalities, the number of enrolled studies and population is pretty small in the meta-analysis, especially for the CT treatment group. There are also no prospective or randomized studies, which would undermine the quality of our study. Secondly, the detailed baseline information is either absent or ununified in a large number of studies, which prevents the more in-depth analysis. It also contributed to the heterogeneity in pooled results. Additionally, although the retrospective study with the information from SEER was conducted, the treatment details were absent.

## Conclusion

This study compared the available treatment modalities efficacy of cAS with meta-analysis of observational studies and summarized data from SEER program. The CT could be inappropriate in localized patients. For metastatic patients, the surgery combined RT was recommended compared with surgery alone since its enhanced OS prognosis. Further investigations of long-term and prospective studies are needed for more solid evidence, especially for those newly developed therapies.

## Data Availability Statement

The original contributions presented in the study are included in the article/**Supplementary Material**. Further inquiries can be directed to the corresponding authors.

## Author Contributions

Conception and design: YC, JC, and SB. Administrative support: YC and JC. Collection and assembly of data: SB and SC. Data analysis and interpretation: SB, SC, and BW. Article writing: SB, SC, and BW. All authors contributed to the article and approved the submitted version.

## Funding

Sichuan Science and Technology Program, Grant number: 2020YFS0267.

## Conflict of Interest

The authors declare that the research was conducted in the absence of any commercial or financial relationships that could be construed as a potential conflict of interest.
