# Supplementary material for: The Effectiveness of Different Treatment Modalities of Cutaneous Angiosarcoma: Results From Meta-Analysis and Observational Data From SEER Database
Source: Front Oncol. 2021 Feb 25;11:627113. doi: 10.3389/fonc.2021.627113 (PMC7947850; doi:10.3389/fonc.2021.627113)
Supplement: Supplementary file 1 [file Table_1.docx]

Supplemental Table 1. “Basic characteristics of included studies.”

| Studies | Year | Number(total) | Gender(male/female) | Tumor size(≤5cm/>5cm) | Tumor site | Tumor grade/stage | Follow-up time(year)^a^ | Treatment |
| --- | --- | --- | --- | --- | --- | --- | --- | --- |
| Hodgkinson, D. J.(1) | 1979 | 13 | 7/6 | / | Scalp: 3; Face: 3;  Temple: 2; Nose: 1;  Lower lip: 1; Left ear: 1;  Scalp and forehead and temple: 2 | / | 8 | Surgery: 5  RT: 3  Surgery and RT: 5 |
| Matsumoto, K.(2) | 1986 | 69 | 43/26 | 13/26  NA:21  (in the scalp and face group, 60 cases) | Scalp and face: 60;  Trunk and limb: 7;  Multiple sites: 2 | Stages I-III: 61;  Stage IV: 8 | 5 | Surgery: 8  RT: 11  CT: 2  Surgery and RT: 7  Surgery and CT: 8  Surgery and RT and CT: 15  RT and CT: 13  NA: 5 |
| Holden, C. A.(3) | 1987 | 72 | 44/28 | / | / | / | 10 | Surgery:7  Surgery and RT: 3  Primary therapy (RT): 45  Primary therapy (CT): 1  No therapy: 7  NA: 9 |
| Barttelbort, S. W.(4) | 1989 | 5 | 4/1 | / | Scalp: 5 | / | 4 | Surgery and RT: 5 |
| Morrison, W. H.(5) | 1995 | 14 | 8/6 | / | Scalp and forehead: 8;  Scalp: 3; Nose: 1;  Infraorbital: 1; Malar skin: 1 | / | / | Only RT: 3  RT and other treatments: 11 |
| Sasaki, R.(6) | 2002 | 30 | 20/10 | 14/16 | Scalp: 21;  Face: 3;  Other sites: 6 | / | 13 | RT: 4  Surgery and RT: 6  RT and IT: 17  Surgery and RT and IT: 3 |
| Pawlik, T. M.(7) | 2003 | 29 | 18/11 | 18/11 | Scalp: 29 | Low grade: 6  High grade: 19  NA: 4 | 8.8 | Primary therapy: (surgery): 28  Primary therapy (RT): 1  Postoperative radiotherapy: 23  Postoperative chemotherapy: 8 |
| Nagano, T.(8) | 2007 | 9 | 4/5 | / | Scalp: 6;  Face: 1;  Neck: 1;  Lower limb: 1 | / | 1.8 | \| CT: 1  Surgery and CT: 1  RT and CT: 1  Surgery and IT and CT: 1  Surgery and RT and IT and CT: 2  RT and IT and CT: 3 \| \| --- \| |
| DeMartelaere, S. L.(9) | 2008 | 21 | 16/5 | 9/6  NA: 6 | Scalp: 5;  Face: 16 | / | 21 | \| Surgery and RT :3  Surgery and CT: 1  Surgery and RT and CT: 2  CTX and surgery: 9  CTX and surgery and RT: 6 \| \| --- \| |
| Köhler, H. F.(10) | 2008 | 23 | 11/12 | 15/8 | Scalp: 9;  Face: 5;  Neck: 9 | Low grade: 5  High grade: 18 | 9 | \| Surgery and RT: 19  Surgery and CT: 2  Surgery and RT and CT: 1  RT and surgery and CT: 1 \| \| --- \| |
| Donghi, D.(11) | 2010 | 9 | 6/3 | / | / | / | 13 | \| Surgery: 2  RT: 1  CT: 1  Surgery and RT: 1  RT and CT: 2  Surgery and RT and CT: 1  No therapy: 1 \| \| --- \| |
| Wollina, U.(12) | 2011 | 8 | 4/4 | / | Head: 5;  Breast: 1;  Lower limb: 2 | pT3N1M0: 1 pT3N0M0: 1 pT1cN0M0: 1  pT2bN0M0: 1 pT2bN1M0: 1 pT2bN0M0: 1  pT2aN0M0: 1 pT1bN0M0 and pT2bN0M0: 1 | 3.3 | Surgery: 3  RT: 2  Surgery and RT: 1  CT and RT: 2 |
| Guadagnolo, B. A.(13) | 2011 | 70 | 50/20 | 49/11  NA: 10 | Scalp: 39;  Face: 30;  Ear: 1 | / | 40 | \| Surgery: 20 \| \| --- \| \| RT: 27 \| \| Surgery and RT: 23 \| |
| Ogawa, K.(14) | 2012 | 48 | 29/19 | 22/26 | Scalp: 46;  Face: 2 | / | 8.8 | \| RT: 5  IT: 4  Surgery and RT: 1  CT and RT: 5  RT and IT: 10  Surgery and RT and CT: 2  Surgery and RT and IT: 9  Surgery and CT and IT: 2  CT and RT and IT: 5  Surgery and RT and CT and IT: 5 \| \| --- \| |
| Perez, M. C.(15) | 2013 | 88 | 38/50 | 48/40 | Head and neck: 38;  Trunk: 13;  RT induced: 30;  Stewart–Treves: 7 | / | 5 | Surgery: 36  RT: 7  CT: 4  RT and CT: 41 |
| Fujisawa, Y.(16) | 2014 | 13 | 8/5 | / | Scalp: 10;  Lower limb: 3 | / | 4 | \| Surgery: 3  CT: 3  Surgery and RT: 2  CT and RT: 2  Surgery and RT and CT: 3 \| \| --- \| |
| Dettenborn, T.(17) | 2014 | 80 | 50/30 | 36/43  NA: 1 | Scalp: 35;  Face: 45 | Grade1: 13  Grade2: 20  Grade3: 1  NA: 46 | 20 | \| surgery: 32 \| \| --- \| \| Surgery and RT: 38 \| \| Surgery and CT: 4 \| \| Surgery and RT and CT: 6 \| |
| Choi, J. H.(18) | 2015 | 14 | 13/1 | / | / | / | 7 | \| Surgery: 3 \| \| --- \| \| Surgery and RT :3 \| \| Surgery and CT :3 \| \| Surgery and CT and RT :5 \| |
| Gründahl, J. E.(19) | 2015 | 103 | 65/38 | 44/59 | Scalp: 26;  Face: 54;  Scalp and face: 23 | / | 11.6 | \| Surgery: 34  RT: 3  CT: 1  Surgery and RT: 48  Surgery and CT: 6  Surgery and RT and CT: 9  RT and CT: 2 \| \| --- \| |
| Patel, S. H.(20) | 2015 | 55 | 39/16 | 20/14  NA: 21 | Scalp: 35;  Face: 20 | Low grade: 12  High grade: 23  NA: 20 | 19 | \| Surgery: 8  RT: 1  CT: 5  Surgery and RT: 18  Surgery and CT: 4  RT and CT: 4  Surgery and CT and RT: 14  No therapy: 1 \| \| --- \| |
| Mullins, B.(21) | 2015 | 6 | 5/1 | 2/3  NA:1 | Scalp: 2; Face: 2;  Larynx and hypopharynx: 1;  Maxillary sinus: 1 | Low and Intermediate grade: 3  High grade: 3 | 6.3 | \| Surgery: 1  RT: 1  CT: 1  Surgery and RT: 2  CT and surgery: 1 \| \| --- \| |
| Ito, T.(22) | 2016 | 43 | 25/18 | 22/21 | Head: 43 | TNM I:23 TNM II: 15 TNM III: 2 TNM IV: 3 | 22 | Taxane: 19  Without taxane: 24 |
| Ogata, D.(23) | 2016 | 5 | 2/3 | / | Scalp: 4;  Back: 1 | / | 1 | \| Surgery and CT: 1 \| \| --- \| \| RT and CT: 3 \| \| Surgery and RT and CT: 1 \| |
| Kitamura, S.(24) | 2017 | 8 | 6/2 | / | Scalp: 8 | / | 2.2 | \| Pazopanib-treated：8  No pazopanib-treated: 10 \| \| --- \| |
| Camarero-Mulas, C.(25) | 2017 | 8 | 7/1 | / | Scalp: 6;  Other sites: 2 | / | 10.8 | Surgery and RT: 5  NA: 3 |
| Chow, T. L.(26) | 2018 | 17 | 12/5 | / | Scalp: 11;  Face: 4;  Scalp and face: 2 | T1N0M0: 6; T1N1M0: 1  T2N0M0: 4; T2N1M0: 3  T2N0M1: 1; T2N1M1: 1 | 3.5 | Surgery: 6  RT: 5  CT: 3  Surgery and RT: 1  No therapy: 2 |
| Oashi, K.(27) | 2018 | 38 | 26/12 | 9/29 | / | / | 8.3 | \| Surgery: 29 \| \| --- \| \| No surgery: 9 \| \| RT: 29 \| \| No RT: 9 \|   CT: 12  No CT: 26  Combined therapy: 23  Single therapy: 15 |
| Campana, L. G.(28) | 2019 | 20 | 7/13 | / | Scalp and face: 7;  Breast and trunk: 10;  Limb: 3 | High grade: 19  NA: 1 | 1.25 | \| ECT: 15  ECT and surgery :2  ECT and surgery and CT :3 \| \| --- \| |
| Zhang, Y.(29) | 2019 | 42 | 25/17 | 19/23 | Scalp: 42 | Low grade: 12 High grade: 30 | 5 | Surgery: 12  Surgery and RT: 6  Surgery and CT: 14  Surgery and RT and CT: 7  Target therapy (apatinib): 3 |
| Wollina, U.(30) | 2019 | 8 | 5/3 | 3/5 | Scalp: 5;  Face: 3 | pT1N0M0: 1 pT2N0M0: 3 pT3N0M0: 1  pT4N0M0: 1 pT4N1M0: 2 | 6.7 | RT: 2  Surgery and RT: 3  CT and RT: 3 |
| Fujisawa, Y.(31) | 2020 | 25 | 21/4 | / | Scalp: 23;  Face: 1;  Other sites: 1 | / | 0.5 | Eribulin mesylate  as 2nd-line chemotherapy: 20  Eribulin mesylate  as 3rd-line or more: 5 |
| Chang, C.(32) | 2020 | 421 | 298/123 | 182/91  NA: 148 | Scalp and neck: 252  Face: 149  Eyelid: 11  External ear: 6  Lip: 3 | High grade: 145  Low grade: 73  NA: 203 | 11.7 | Surgery: 138  Surgery and RT: 178  Surgery RT and CT: 48  RT and CT: 28  Surgery and CT: 29 |

CTX: Neoadjuvant chemotherapy

ECT: Electrochemotherapy

^a^: Mean longest follow-up time

1. Hodgkinson DJ, Soule EH, Woods JE. Cutaneous angiosarcoma of the head and neck. *Cancer* (1979) 44(3):1106-13. Epub 1979/09/01. doi: 10.1002/1097-0142(197909)44:3<1106::aid-cncr2820440345>3.0.co;2-c.

2. Matsumoto K, Inoue K, Fukamizu H. *Prognosis of cutaneous angiosarcoma in Japan: A statistical study of sixty-nine cases*: Chirurgia Plastica. 8 (3) (pp 151-158), 1986. Date of Publication: 1986.

3. Holden CA, Spittle MF, Jones EW. Angiosarcoma of the face and scalp, prognosis and treatment. *Cancer* (1987) 59(5):1046-57. Epub 1987/03/01. doi: 10.1002/1097-0142(19870301)59:5<1046::aid-cncr2820590533>3.0.co;2-6.

4. Barttelbort SW, Stahl R, Ariyan S. Cutaneous angiosarcoma of the face and scalp. *Plastic and reconstructive surgery* (1989) 84(1):55-9. Epub 1989/07/01. doi: 10.1097/00006534-198907000-00011.

5. Morrison WH, Byers RM, Garden AS, Evans HL, Ang KK, Peters LJ. Cutaneous angiosarcoma of the head and neck. A therapeutic dilemma. *Cancer* (1995) 76(2):319-27.

6. Sasaki R, Soejima T, Kishi K, Imajo Y, Hirota S, Kamikonya N, et al. Angiosarcoma treated with radiotherapy: impact of tumor type and size on outcome. *International journal of radiation oncology, biology, physics* (2002) 52(4):1032-40. Epub 2002/04/18. doi: 10.1016/s0360-3016(01)02753-5.

7. Pawlik TM, Paulino AF, McGinn CJ, Baker LH, Cohen DS, Morris JS, et al. Cutaneous angiosarcoma of the scalp: a multidisciplinary approach. *Cancer* (2003) 98(8):1716-26.

8. Nagano T, Yamada Y, Ikeda T, Kanki H, Kamo T, Nishigori C. Docetaxel: a therapeutic option in the treatment of cutaneous angiosarcoma: report of 9 patients. *Cancer* (2007) 110(3):648-51.

9. DeMartelaere SL, Roberts D, Burgess MA, Morrison WH, Pisters PW, Sturgis EM, et al. Neoadjuvant chemotherapy-specific and overall treatment outcomes in patients with cutaneous angiosarcoma of the face with periorbital involvement. *Head Neck* (2008) 30(5):639-46.

10. Kohler HF, Neves RI, Brechtbuhl ER, Mattos Granja NV, Ikeda MK, Kowalski LP. Cutaneous angiosarcoma of the head and neck: report of 23 cases from a single institution. *Otolaryngology Head & Neck Surgery* (2008) 139(4):519-24.

11. Donghi D, Kerl K, Dummer R, Schoenewolf N, Cozzio A. Cutaneous angiosarcoma: own experience over 13 years. Clinical features, disease course and immunohistochemical profile. *J Eur Acad Dermatol Venereol* (2010) 24(10):1230-4.

12. Wollina U, Hansel G, Schonlebe J, Averbeck M, Paasch U, Uhl J, et al. Cutaneous angiosarcoma is a rare aggressive malignant vascular tumour of the skin. *Journal of the European Academy of Dermatology & Venereology* (2011) 25(8):964-8.

13. Guadagnolo BA, Zagars GK, Araujo D, Ravi V, Shellenberger TD, Sturgis EM. Outcomes after definitive treatment for cutaneous angiosarcoma of the face and scalp. *Head & neck* (2011) 33(5):661-7. Epub 2010/10/21. doi: 10.1002/hed.21513.

14. Ogawa K, Takahashi K, Asato Y, Yamamoto Y, Taira K, Matori S, et al. Treatment and prognosis of angiosarcoma of the scalp and face: a retrospective analysis of 48 patients. *The British journal of radiology* (2012) 85(1019):e1127-33. Epub 2012/07/19. doi: 10.1259/bjr/31655219.

15. Perez MC, Padhya TA, Messina JL, Jackson RS, Gonzalez RJ, Bui MM, et al. Cutaneous angiosarcoma: a single-institution experience. *Ann Surg Oncol* (2013) 20(11):3391-7.

16. Fujisawa Y, Nakamura Y, Kawachi Y, Otsuka F. Comparison between taxane-based chemotherapy with conventional surgery-based therapy for cutaneous angiosarcoma: a single-center experience. *J Dermatolog Treat* (2014) 25(5):419-23.

17. Dettenborn T, Wermker K, Schulze HJ, Klein M, Schwipper V, Hallermann C. Prognostic features in angiosarcoma of the head and neck: a retrospective monocenter study. *Journal of cranio-maxillo-facial surgery : official publication of the European Association for Cranio-Maxillo-Facial Surgery* (2014) 42(8):1623-8. Epub 2014/06/26. doi: 10.1016/j.jcms.2014.05.002.

18. Choi JH, Ahn KC, Chang H, Minn KW, Jin US, Kim BJ. Surgical Treatment and Prognosis of Angiosarcoma of the Scalp: A Retrospective Analysis of 14 Patients in a Single Institution. *BioMed research international* (2015) 2015:321896. Epub 2015/12/30. doi: 10.1155/2015/321896.

19. Grundahl JE, Hallermann C, Schulze HJ, Klein M, Wermker K. Cutaneous Angiosarcoma of Head and Neck: A New Predictive Score for Locoregional Metastasis. *Translational Oncology* (2015) 8(3):169-75.

20. Patel SH, Hayden RE, Hinni ML, Wong WW, Foote RL, Milani S, et al. Angiosarcoma of the scalp and face: the Mayo Clinic experience. *JAMA otolaryngology-- head & neck surgery* (2015) 141(4):335-40. Epub 2015/01/31. doi: 10.1001/jamaoto.2014.3584.

21. Mullins B, Hackman T. Angiosarcoma of the Head and Neck. *International archives of otorhinolaryngology* (2015) 19(3):191-5. Epub 2015/07/15. doi: 10.1055/s-0035-1547520.

22. Ito T, Uchi H, Nakahara T, Tsuji G, Oda Y, Hagihara A, et al. Cutaneous angiosarcoma of the head and face: a single-center analysis of treatment outcomes in 43 patients in Japan. *J Cancer Res Clin Oncol* (2016) 142(6):1387-94.

23. Ogata D, Yanagisawa H, Suzuki K, Oashi K, Yamazaki N, Tsuchida T. Pazopanib treatment slows progression and stabilizes disease in patients with taxane-resistant cutaneous angiosarcoma. *Medical oncology (Northwood, London, England)* (2016) 33(10):116. Epub 2016/09/11. doi: 10.1007/s12032-016-0831-z.

24. Kitamura S, Yanagi T, Inamura Y, Hata H, Imafuku K, Yoshino K, et al. Pazopanib does not bring remarkable improvement in patients with angiosarcoma. *The Journal of dermatology* (2017) 44(1):64-7. Epub 2016/08/30. doi: 10.1111/1346-8138.13558.

25. Camarero-Mulas C, Martinez-Mera C, Capusan TM, Rodriguez-Jimenez P, Navarro-Tejedor R, Urquia-Renke A, et al. *Cutaneous angiosarcoma of the head and neck: A restrospective analysis*: Journal of the American Academy of Dermatology. Conference: 75th Annual Meeting of the American Academy of Dermatology. United States. 76 (6 Supplement 1) (pp AB88), 2017. Date of Publication: June 2017.

26. Chow TL, Kwan WW, Kwan CK. Treatment of cutaneous angiosarcoma of the scalp and face in Chinese patients: local experience at a regional hospital in Hong Kong. *Hong Kong Med J* (2018) 24(1):25-31.

27. Oashi K, Namikawa K, Tsutsumida A, Takahashi A, Itami J, Igaki H, et al. Surgery with curative intent is associated with prolonged survival in patients with cutaneous angiosarcoma of the scalp and face -a retrospective study of 38 untreated cases in the Japanese population. *European journal of surgical oncology : the journal of the European Society of Surgical Oncology and the British Association of Surgical Oncology* (2018) 44(6):823-9. Epub 2018/03/21. doi: 10.1016/j.ejso.2018.02.246.

28. Campana LG, Kis E, Bottyan K, Orlando A, de Terlizzi F, Mitsala G, et al. Electrochemotherapy for advanced cutaneous angiosarcoma: A European register-based cohort study from the International Network for Sharing Practices of electrochemotherapy (InspECT). *International Journal Of Surgery* (2019) 72:34-42.

29. Zhang Y, Yan Y, Zhu M, Chen C, Lu N, Qi F, et al. Clinical outcomes in primary scalp angiosarcoma. *Oncol Lett* (2019) 18(5):5091-6.

30. Wollina U, Koch A, Hansel G, Schönlebe J, Lotti T, Vojvodic A. Cutaneous Angiosarcoma of Head and Neck - A Single-Centre Analysis. *Open Access Maced J Med Sci* (2019) 7(18):2976-8.

31. Fujisawa Y, Fujimura T, Matsushita S, Yamamoto Y, Uchi H, Otsuka A, et al. The efficacy of eribulin mesylate for patients with cutaneous angiosarcoma previously treated with taxane: a multi-center, prospective, observational study. *Br J Dermatol* (2020) 20(10):19042.

32. Chang C, Wu SP, Hu K, Li Z, Schreiber D, Oliver J, et al. Patterns of Care and Survival of Cutaneous Angiosarcoma of the Head and Neck. *Otolaryngol Head Neck Surg* (2020) 11(194599820905495):0194599820905495.
