# Supplementary material for: The Effectiveness of Different Treatment Modalities of Cutaneous Angiosarcoma: Results From Meta-Analysis and Observational Data From SEER Database
Source: Front Oncol. 2021 Feb 25;11:627113. doi: 10.3389/fonc.2021.627113 (PMC7947850; doi:10.3389/fonc.2021.627113)
Supplement: Supplementary file 3 [file Table_3.docx]

Supplementary table 3. Univariate cox proportions hazards models for overall survival (OS) and cancer specific survival (CSS) in SEER patients with cAS.

|  | OS | | | CSS | | |
| --- | --- | --- | --- | --- | --- | --- |
|  | HR | p value | HR 95% CI | HR | p value | HR 95% CI |
| Age |  |  |  |  |  |  |
| 80+ | Ref |  |  |  |  |  |
| 10-39 | 0.07 | 0.00 | (0.03,0.2) | 0.18 | 0.02 | (0.04,0.74) |
| 40-49 | 0.22 | 0.00 | (0.13,0.39) | 0.61 | 0.15 | (0.3,1.21) |
| 50-59 | 0.21 | 0.00 | (0.15,0.31) | 0.37 | 0.00 | (0.22,0.65) |
| 60-69 | 0.40 | 0.00 | (0.32,0.51) | 0.53 | 0.00 | (0.37,0.77) |
| 70-79 | 0.58 | 0.00 | (0.48,0.7) | 0.61 | 0.00 | (0.45,0.84) |
| Sex |  |  |  |  |  |  |
| Female | Ref |  |  |  |  |  |
| Male | 1.11 | 0.18 | (0.95,1.31) | 1.47 | 0.00 | (1.13,1.91) |
| Race |  |  |  |  |  |  |
| White | Ref |  |  |  |  |  |
| Black | 1.69 | 0.00 | (1.2,2.37) | 1.56 | 0.14 | (0.87,2.8) |
| Other | 1.30 | 0.11 | (0.94,1.81) | 2.37 | 0.00 | (1.55,3.62) |
| Unknown | 0.37 | 0.05 | (0.14,0.99) | 0.52 | 0.36 | (0.13,2.1) |
| Sites |  |  |  |  |  |  |
| Scalp/neck/head | Ref |  |  |  |  |  |
| Face | 0.62 | 0.00 | (0.5,0.76) | 0.50 | 0.00 | (0.37,0.68) |
| Trunk/limb | 0.57 | 0.00 | (0.47,0.69) | 0.13 | 0.00 | (0.09,0.21) |
| Unspecific site | 1.39 | 0.20 | (0.84,2.3) | 1.30 | 0.48 | (0.64,2.64) |
| SEER historic stage |  |  |  |  |  |  |
| Distant | Ref |  |  |  |  |  |
| Localized | 0.63 | 0.00 | (0.53,0.75) | 0.57 | 0.00 | (0.43,0.75) |
| Unstaged | 0.82 | 0.11 | (0.65,1.05) | 0.64 | 0.04 | (0.42,0.97) |
| Histologic grade |  |  |  |  |  |  |
| Grade I | Ref |  |  |  |  |  |
| Grade II | 1.15 | 0.54 | (0.74,1.8) | 0.97 | 0.93 | (0.44,2.1) |
| Grade III | 1.82 | 0.00 | (1.21,2.72) | 1.73 | 0.11 | (0.88,3.4) |
| Grade IV | 1.77 | 0.01 | (1.18,2.66) | 1.78 | 0.09 | (0.91,3.5) |
| Unknown | 1.62 | 0.01 | (1.12,2.35) | 1.75 | 0.07 | (0.95,3.24) |
| Size |  |  |  |  |  |  |
| >5cm | Ref |  |  |  |  |  |
| <5cm | 0.22 | 0.03 | (0.06,0.89) | - | - | - |
| NA/Not reported | 1.09 | 0.31 | (0.92,1.29) | - | - | - |

HR: hazard ratio; CI: confidential interval; OS: overall survival; CSS: cancer-specific survival; The size was excluded in the analysis model for CSS since its overfitting effect.
