# Supplementary material for: The Effectiveness of Different Treatment Modalities of Cutaneous Angiosarcoma: Results From Meta-Analysis and Observational Data From SEER Database
Source: Front Oncol. 2021 Feb 25;11:627113. doi: 10.3389/fonc.2021.627113 (PMC7947850; doi:10.3389/fonc.2021.627113)
Supplement: Supplementary file 4 [file Table_4.docx]

Supplementary table 4. Multivariate cox proportions hazards models for overall survival (OS) and cancer-specific survival (CSS) in SEER patients with cAS.

|  | Localized |  |  |  |  |  | Metastatic | |  |  |  |  |
| --- | --- | --- | --- | --- | --- | --- | --- | --- | --- | --- | --- | --- |
|  | OS |  |  | CSS |  |  | OS |  |  | CSS |  |  |
|  | HR | 95% CI | P value | HR | 95% CI | P value | HR | 95% CI | P value | HR | 95% CI | P value |
| Age |  |  |  |  |  |  |  |  |  |  |  |  |
| 80+ | Ref |  |  |  |  |  |  |  |  |  |  |  |
| 10-39 | 0.15 | (0.05,0.45) | <0.01 | 0.92 | (0.2,4.15) | 0.91 | - | - | - | - | - | - |
| 40-49 | 0.2 | (0.09,0.43) | <0.01 | 0.41 | (0.14,1.19) | 0.1 | - | - | - | - | - | - |
| 50-59 | 0.24 | (0.14,0.41) | <0.01 | 0.54 | (0.25,1.14) | 0.11 | - | - | - | - | - | - |
| 60-69 | 0.36 | (0.25,0.53) | <0.01 | 0.38 | (0.2,0.72) | <0.01 | - | - | - | - | - | - |
| 70-79 | 0.54 | (0.4,0.72) | <0.01 | 0.39 | (0.23,0.66) | <0.01 | - | - | - | - | - | - |
| Sex* |  |  |  |  |  |  |  |  |  |  |  |  |
| Female | Ref |  |  |  |  |  |  |  |  |  |  |  |
| Male | - | - | - | - | - | - | 0.99 | (0.7,1.39) | 0.93 | 0.67 | (0.41,1.08) | 0.1 |
| Race* |  |  |  |  |  |  |  |  |  |  |  |  |
| White | Ref |  |  |  |  |  |  |  |  |  |  |  |
| Black | 1.62 | (0.87,3.01) | 0.13 | - | - | - | 1.22 | (0.7,2.12) | 0.48 | 0.97 | (0.41,2.26) | 0.94 |
| Other | 1.28 | (0.79,2.06) | 0.31 | - | - | - | 1.09 | (0.55,2.17) | 0.8 | 1.37 | (0.59,3.19) | 0.46 |
| Unknown | 0.37 | (0.05,2.68) | 0.33 | - | - | - | 0.33 | (0.04,2.44) | 0.28 | 0.49 | (0.06,3.83) | 0.49 |
| Sites |  |  |  |  |  |  |  |  |  |  |  |  |
| Scalp/neck/head | Ref |  |  |  |  |  |  |  |  |  |  |  |
| Face | 0.58 | (0.43,0.8) | <0.01 | 0.33 | (0.2,0.55) | <0.01 | 0.63 | (0.43,0.92) | 0.02 | 0.77 | (0.46,1.3) | 0.33 |
| Trunk/limb | 0.69 | (0.51,0.95) | 0.02 | 0.16 | (0.08,0.32) | <0.01 | 0.43 | (0.28,0.65) | <0.01 | 0.11 | (0.05,0.24) | <0.01 |
| Unspecific site | 0.67 | (0.29,1.57) | 0.36 | 1 | (0.35,2.86) | 1 | 1.36 | (0.62,2.99) | 0.44 | 0.67 | (0.21,2.09) | 0.49 |
| Histologic grade | |  |  |  |  |  |  |  |  |  |  |  |
| Grade I | Ref |  |  |  |  |  |  |  |  |  |  |  |
| Grade II | 1.29 | (0.69,2.41) | 0.43 | 0 | 2.27 | (0.59,8.8) | 0.8 | (0.37,1.75) | 0.58 | 0.52 | (0.15,1.82) | 0.31 |
| Grade III | 1.69 | (0.93,3.06) | 0.09 | 0.86 | 2.71 | (0.77,9.53) | 0.93 | (0.5,1.7) | 0.81 | 0.56 | (0.22,1.42) | 0.22 |
| Grade IV | 1.72 | (0.95,3.1) | 0.07 | 1.28 | 2.54 | (0.72,8.91) | 1.22 | (0.66,2.28) | 0.53 | 1.04 | (0.41,2.64) | 0.93 |
| Unknown | 1.62 | (0.95,2.79) | 0.08 | 1.67 | 2.97 | (0.9,9.73) | 0.97 | (0.54,1.72) | 0.9 | 0.59 | (0.25,1.4) | 0.23 |
| Size* |  |  |  |  |  |  |  |  |  |  |  |  |
| >5cm | Ref |  |  |  |  |  |  |  |  |  |  |  |
| <5cm | - | - | - | - | - | - | - | - | - | - | - | - |
| NA/Not reported | - | - | - | - | - | - | - | - | - | - | - | - |
| Treatment modality | |  |  |  |  |  |  |  |  |  |  |  |
| Surgery and RT | Ref |  |  |  |  |  |  |  |  |  |  |  |
| CT | 3.6 | (1.95,6.62) | <0.01 | 3.16 | (1.17,8.53) | 0.02 | 4.17 | (2.05,8.46) | <0.01 | 3.53 | (1.24,10.02) | 0.02 |
| None | 1.72 | (1.01,2.91) | 0.05 | 1.83 | (0.83,4.05) | 0.13 | 4.36 | (2.3,8.25) | <0.01 | 6.78 | (2.77,16.59) | <0.01 |
| RT | 1.62 | (0.94,2.77) | 0.08 | 0.9 | (0.36,2.24) | 0.81 | 1.61 | (0.83,3.11) | 0.16 | 1.37 | (0.52,3.62) | 0.53 |
| RT+CT | 1.61 | (0.82,3.15) | 0.16 | 1.17 | (0.44,3.13) | 0.75 | 1.11 | (0.54,2.25) | 0.78 | 1.82 | (0.76,4.33) | 0.18 |
| Surgery | 0.99 | (0.71,1.4) | 0.98 | 0.62 | (0.36,1.07) | 0.08 | 1.6 | (1.05,2.42) | 0.03 | 1.15 | (0.62,2.15) | 0.66 |
| Surgery and CT | 2.25 | (1.19,4.24) | 0.01 | 1.71 | (0.57,5.12) | 0.34 | 1.18 | (0.67,2.06) | 0.56 | 1.04 | (0.46,2.39) | 0.92 |
| Surgery and RT and CT | 1.79 | (1.03,3.13) | 0.04 | 1.89 | (0.88,4.04) | 0.1 | 1.3 | (0.72,2.33) | 0.38 | 1.87 | (0.91,3.86) | 0.09 |

* the covariate was excluded in the analysis since the overfitting effect.

OS: overall survival; CSS: cancer-specific survival; CT: chemotherapy; RT: radiotherapy; HR: hazard ratio; CI: confidential interval; NA: not available.
